# Unique Theory of Mind Differentiation in Children with Autism and Asperger Syndrome

**DOI:** 10.1155/2012/505393

**Published:** 2012-04-05

**Authors:** Michele Tine, Joan Lucariello

**Affiliations:** ^1^Department of Education, Dartmouth College, 6103 Raven House, Hanover, NH 03755, USA; ^2^Office of Academic Affairs, The City University of New York, 535 East 80th Street, NY 10021, USA

## Abstract

This study was designed to determine if ToM abilities of children with autism and Asperger syndrome differentiate into Intrapersonal ToM and Social ToM. A battery of Social and Intrapersonal ToM tasks was administered to 39 children with autism and 34 children with Asperger syndrome. For both groups of children, ToM differentiated and Intrapersonal ToM was stronger than Social ToM. This asymmetry was greater for children with autism, whose Social ToM was especially weak. These results support a differentiated, as opposed to integrated, ToM. Moreover, the findings provide a more thorough understanding of the cognitive abilities associated with autism and Asperger syndrome.

## 1. Introduction

Theory of Mind (ToM) entails our imputation of mental states to the self and to others to account for behavior. A foundational question about ToM is whether it is a single unitary construct or differentiates into separable abilities. The predominant accounts of ToM, which are outlined in Table 1, most often advocate an integrated view wherein reasoning about the mental states of self and others are deemed to be one and the same cognitive ability.

More specifically, the module proposed by the *modularity* account is said to automatically compute the mental states of self and others. Similarly, according to *theory-theory*, the conceptual change that occurs during the replacement of successive theories does not distinguish between the mental states of self and others. The *sociocultural* account assumes that the social contextual variables that drive ToM development equally affect the development of reasoning about self and others' mental states. Finally, the *language* account does not assess if the relationship between aspects of language differs across self and other reasoning.

Two theories that refute the integrated view in favor of a differentiated view are the *Functional Multilinear Socialization* (FMS)* Model* and *simulation* theory. These accounts of ToM distinguish reasoning of one's own and others' mental states as two distinct cognitive abilities that are not purported to emerge necessarily together at a single ontogenetic point in time. The FMS Model poses that ToM capabilities differentiate into the two distinct cognitive abilities of Social Reasoning (reasoning about *others'* mental states) and Intrapersonal Reasoning (reasoning about one's *own* mental states) [1, 2]. Moreover, the FMS Model defines Social and Intrapersonal Reasoning in relation to the everyday functions they each play. The Social Reasoning component of ToM is viewed as critically important in social interactions. To carry out successful social interactions, one must be able to understand and predict the mental states of other people, especially when these mental states are inconsistent with reality. The FMS Model is also able to capture a distinct Intrapersonal Reasoning use of ToM-learning. Learning involves the representational change of one's own mental representations. Based on these functional uses, The FMS Model can account for the possibility that children may develop strengths in Social or Intrapersonal ToM.

Simulation theory is the other differentiated account of ToM and proposes that a person initially has a more accurate and advanced reasoning about their own mental states and then uses those advanced self-reasoning skills as a map on which to simulate other's reasoning [3, 4]. Accordingly, Intrapersonal ToM is always stronger than Social ToM functioning, as simulation theory assigns primacy to reasoning about one's own representations.

Previous research with typically developing populations elucidates that ToM capabilities do in fact differentiate into Social and Intrapersonal constructs. Performance across false belief (which assesses Social ToM) and appearance-reality tasks (which assesses Intrapersonal ToM) is not correlated [5]. Similarly, Moore and colleagues [6] found performance across false belief and representational change (or “own” belief) not to be correlated. Ruffman and colleagues' [7] results also support a differentiated ToM model; the majority of their 5-year olds passed an “other” false belief task, but failed a source task (that assessed a child's understanding of the source of their own representations.) Cutting and Dunn [8] presented participants with eight false belief tasks, one of which was designed to be a “recall your own” false belief. This “own” task was the only task of the eight not correlated with the others.

Moreover, studies designed to specifically test the FMS Model [1, 2] have supported differentiated ToM. In 2004, Lucariello found that performance across social and intrapersonal tasks was not correlated in a sample of low-income 5-6-year olds. Lucariello et al. [2] showed that ToM performance differentiated and was better in the social than intrapersonal condition with low- and middle-SES kindergarten students. In addition, Butler and laucariallo [9] found evidence of uneven development of Social and Intrapersonal ToM in gifted children, who performed better on intrapersonal than social tasks.

Integrated accounts of ToM cannot account for these data. The fact that ToM differentiates on social and intrapersonal tasks provides compelling evidence that ToM is composed of the distinct abilities of Social and Intrapersonal Reasoning. According to simulation theory, Intrapersonal ToM should always be stronger than Social ToM functioning a suggestion that goes against some of the aforementioned data showing some children exhibit a relative strength in Social ToM [2, 9]. However, the FMS Model [1, 2] can account for the fact that children may perform differently on social and intrapersonal tasks and show asymmetric development in both directions with some children showing that a strength in Social Reasoning [1, 2] and others a strength in Intrapersonal Reasoning [9].

Admittedly, Wellman et al. [10] meta analysis suggests that there are no differences between own and other reasoning tasks. However, this analysis was run only on own and other reasoning within a false belief task paradigm. Many other tasks can be used to measure one's own reasoning such as appearance reality, source, and emotion vignette tasks [7, 11]. It seems possible that own and other reasoning do not differentiate within the constraints of a false belief task, but do so on other assessments (as supported by the more recent aforementioned studies that were not included in the meta-analysis).

It is important to determine the nature of ToM differentiation in children with autism, as much attention has been given to the hypothesis that autism entails a disturbance in ToM. It has been suggested that this disturbance may underlie and account for the wide range of known symptoms associated with the disorder. However, thus far, research has been conducted primarily under the assumption that ToM is an integrated ability. Studies testing false belief, deception, and emotion almost exclusively use measures that only tap the reasoning of *others*, but then generalize to suggest poor reasoning about self and other.

In the 1980s it was discovered that children with autism fail to understand *another* person's false beliefs about the world [12]. Numerous studies have replicated this finding (see [13] for a review). However, none of the tasks directly assessed and/or compared the children's reasoning of their own beliefs and beliefs of others. Deception is also relevant to understanding other minds, as it involves trying to make someone else believe that something is true when it is actually false. It has been found repeatedly that children with autism show difficulties producing and understanding deception [14, 15]. While a lack of deceptive abilities reflects a problem in understanding others' minds, it is not directly related to one's mental processes about oneself. It has been found that children with autism can recognize simple emotions, but have difficulty recognizing and predicting belief based-emotions (specifically, the emotion of surprise) [16, 17]. Yet, these tests only measured this ability using scenarios in which children with autism had to predict the mental state *of a character* as cause of that character's emotion. Again, the currently accepted integrated definition of ToM has caused us to accept that these children have a general inability to understand mental states as a cause, when their ability to determine if their *own* beliefs can cause their own emotions has never been tested.

It is important to note the few studies that have assessed the ability of children with autism to understand mental states of self and self compared to others and have not found significant differences between the two. Admittedly, Kazak et al. [18] asked young people with autism whether they knew or only guessed what was in a box having on some trials seen inside. In a second condition, children were asked if the experimenter knew or only guessed what was in the box. The results showed no superiority in judging own knowledge versus judging other's knowledge. However, as mentioned earlier in regard to the nonautistic population, it certainly seems possible that own and other reasoning do not differentiate within the domain of knowledge/belief, but do so within other domains and/or other tasks that can be used to measure one's own reasoning.

Baron-Cohen's [19] study also investigated self-reasoning. They used an appearance-reality task in which children with autism were shown a misleading object. While nonautistic subjects were able to correctly answer an appearance question “What does it really look like?” and a reality question “What is it really?”, only a small percentage of children with autism were able to do so. Although at the surface appearance reality tasks are about an object, they tap into a participants own beliefs about the object. The study, therefore, provides some evidence that children with autism have difficulty understanding their own mental states (about an object), but does not offer insight into this weakness relative to the weakness of understanding others' mental states.

One study tested the dissociation between self and other reasoning in the autistic population and their results support a *differentiated* ToM. Leekam and perner [20] used a simplified version of the Zaitchik (1990) “false photograph” task (which is modeled on the standard false belief task except insofar as it tests children's ability to reason about physical photographic misrepresentation) with a group of teenagers with high-functioning autism. One condition tested false belief of others. The second condition tested what they refer to as photographic misrepresentations, but can be considered what we term “intrapersonal,” as it tapped the participants own beliefs.

In both conditions, participants were shown a doll (Judy) wearing a red dress. In the false belief condition, a second doll (Susan) sees Judy in the red dress and then leaves the room. Judy's dress is changed from red to green, and subjects are asked “What color does Susan think that Judy's dress is?” In the false photograph condition, a Polaroid photo is taken of Judy in the red dress. While the photo is developing, her dress is again changed from red to green and participants are asked “In the picture, what color is Judy's dress?” This question aims to tap into what the subject themselves believe. Only 25% of participants with autism were correct on the false-belief question, but almost all of those tested passed the false-photograph question [20]. Similar results were obtained by Leslie and Thaiss [21]. Results like this strongly suggest that individuals with autism have a specific inability to reason about the mental states and processes of others. However, they are not generally impaired in their reasoning abilities about self. 

Relatively little research specifically tests the ToM abilities of children with Asperger syndrome, let alone their specific Social and Intrapersonal ToM abilities. Often these children are included in ToM studies, but are folded into samples described as children with autism spectrum disorders (e.g., [28–31]). This may reflect that many researchers do not think of Asperger syndrome as a distinct disorder, but a variant of autism, and located on the milder end of the autism spectrum [32–35]. The few studies that do analyze the ToM abilities of children with autism separately from those with Asperger syndrome have reported inconsistent results. Some suggest deficiencies in ToM abilities are common to both people with autism and Asperger syndrome [36]. Others indicate that these deficits are less characteristic of Asperger syndrome and suggest that this may be a basis on which the two conditions can be distinguished [37, 38]. Nonetheless, all of these studies were based on false belief tasks and were therefore unable to illuminate potential differences across Social and Intrapersonal ToM abilities.

It is also important to note that studies indicating differences in ToM abilities across subjects with autism and Asperger syndrome have often been criticized on the grounds that the findings could be attributable to poor subject matching on language abilities. There has been strong evidence for a positive correlation between verbal skills and ToM abilities [39]. Thus, some argue the apparently better ToM capacity in people with Asperger syndrome may just be a reflection of their higher verbal abilities [40, 41]. More research is needed to determine if ToM differentiation patterns are different between children with Asperger syndrome and autism. Differences in verbal abilities need to be addressed and Social and Intrapersonal ToM need to be specifically measured.

The goal of the present study was threefold. The first was to determine if ToM differentiates into Social ToM and Intrapersonal ToM in children with autism and Asperger syndrome. It is hypothesized that ToM will differentiate. This hypothesis is based on (1) previous research in typically developing populations showing ToM differentiation and (2) studies with these populations reporting (only) impairments in reasoning about others' mental states. The second goal was to determine if children with autism and Asperger syndrome exhibit more severe deficits in Social ToM than Intrapersonal ToM. It is hypothesized that both children with autism and Asperger syndrome will exhibit more severe deficits in Social ToM than Intrapersonal ToM. This hypothesis is based on the fact that both disorders are characterized by social interaction impairments, but not necessarily learning impairments [42] and the FMS Model proposes that Social ToM is used in social interactions and Intrapersonal ToM in learning. The third goal was to determine if ToM differentiates differently for children with autism compared to children with Asperger syndrome. It is hypothesized that children with autism will show an even more severe deficit in Social ToM than children with Asperger syndrome, as the severity of the social interaction impairments is greater in autism [42] and the FMS Model proposes that Social ToM is used in social interactions. There is no clear hypothesis generated regarding the Intrapersonal ToM abilities in children with autism compared to Asperger syndrome, as to date no research has been conducted on the comparative Intrapersonal ToM capabilities of either population. Moreover, the proposed functional use of Intrapersonal ToM (learning) is not necessarily impaired in either disorder [42].

## 2. Methods

### 2.1. Participants

Participants included 73 children drawn from 12 schools in the New England area. Thirty-nine of the participants had autism and 34 had Asperger syndrome. Diagnostic classification was based on reports made by clinical psychologists and/or psychiatrists. Participants classified as having autism met the specific diagnostic criteria for DSM-IV-TR 299.000 “Autistic Disorder,” whereas participants classified as having Asperger's met the specific diagnostic criteria for DSM-IV-TR 299.80 “Asperger's Disorder”. Only participants with detailed qualitative notes documenting the ways in which each diagnostic criterion were (or were not) met were recruited for the study. No participants had concurrent DSM-IV-TR disorder diagnoses. The full sample included 73 males and 8 females (children with autism: 33 males, 6 females; children with Asperger syndrome: 32 males, 2 females.) These numbers reflect the fact that these disorders are much more prevalent in males than females [42]. The mean chronological age of the full sample was 10.4 years, with a range of 8.04−13.0 years (children with autism: 10.3; children with Asperger syndrome: 10.4). The mean mental age of the full sample was 10.4, with a range of 8.0–14.9 (children with autism: 10.2, children with Asperger syndrome: 10.6).

### 2.2. Measures

#### 2.2.1. Intelligence and Mental Age Measure

The Raven's Progressive Matrices (RPMs) [43] was used as an intelligence and mental age measure. An intelligence cutoff score of 70 was established due to the cognitive demands necessary to complete the battery of Theory of Mind tasks proposed. Mental age was calculated based on RPM performance, following guidelines presented in the RPM Manuel Research Supplement 3 [44]. The mental age cutoff of 8 was established to ensure that ToM weaknesses found were not due to general developmental immaturity. Metarepresentational ToM is usually attained around the mental ages of 4-5 for typically developing children (Flavell, Flavell, and Green 1983) [45], but not until at least the mental age of 8-9 for children with autism [46].

#### 2.2.2. Language Measure

The Test of Language Development Intermediate Fourth Edition (TOLD:I-4) [47] was administered to each participant. The TOLD:I-4 measures general language, semantics, receptive vocabulary, and syntax, which have been reported to be related to ToM [27].

#### 2.2.3. Theory of Mind Measure

The battery of ToM tasks consisted of four metarepresentational reasoning tasks that tapped mental state reasoning across the domains of emotions, beliefs, and perceptions. The four tasks were story vignettes, unexpected contents, unexpected identity, and color filters. See Table 2 for the tasks and metarepresentational behaviors they assessed. Each of the four tasks tapped both Social and Intrapersonal ToM reasoning, which represented separate conditions. A within-subject design was employed such that each participant received the four tasks in both the Social and Intrapersonal conditions. Character gender was matched to participant gender.

**Story Vignette Pretest**

Children were also given a pretest to assess their understanding of the emotions used in the story vignette task (happy, sad, okay). Children had to correctly link each emotion to prototypical situations that elicit that emotion. Two memory pretest questions for each story were also administered. One probed recall of the situation that caused the real emotion. The second probed recall of the reason for displaying a different emotion. All participants correctly answered all memory questions.

**Unexpected Contents Pretest**

In both conditions, a pretest question of “What's inside the box?” was administered after the box had been opened, but prior to administration of test questions, to assess that children realize the actual contents of the box. All children passed the pretest.

**Unexpected Identity Pretest**

A pretest question of “What is this object really? Is it really a sponge or is it really a rock?” was administered after the child touched the object, but before the test questions were administered, to assess the child's knowledge that the object's actual identity was a sponge. Four children with autism failed this pretest by providing no response or an inappropriate response and were excluded from analyses.

**Color Filters Pretraining**

Participants received a pretraining phase (Flavell et al. 1986) where the experimenter used a sample object and filter to demonstrate the different colors of an object appeared to the experimenter and child when only the child was looking through a filter.

### 2.3. Procedure

Each child participated in two individual testing sessions. Each session took place in a quiet space outside of the child's classroom and lasted approximately 45 minutes. During the first session, the language and IQ measures were administered along with a Social or Intrapersonal ToM story vignette task. The condition (social, intrapersonal) of the story vignette administered in the first session was counterbalanced across participants. During the second session, the remaining ToM tasks were administered. The order of the remaining tasks was counterbalanced across participants. In addition, the order of the condition first presented within each task was counterbalanced across participants. For example, for the quarter of the participants assigned to receive the unexpected identity task first, half received the intrapersonal questions followed by the social questions and half received the social questions followed by the intrapersonal questions.

### 2.4. Statistical Analyses

The statistical software SPSS 16 was used for all analyses. Descriptive statistics and correlations were obtained for all measures. A series of stepwise linear regressions was used to investigate the contribution of language and IQ on ToM performance. Repeated Measures ANCOVAs were used to determine if Social ToM scores and Intrapersonal ToM scores were significantly different and if they varied as a function of participant group.

## 3. Results

### 3.1. Computing Composite Social and Intrapersonal ToM Scores

The six social tasks were used to compute the composite “Social ToM” score for each participant, as the reliability of these six tasks was moderate (Cronbach's *α* = .64) and measured a variety of mental states (emotions, beliefs, and perception). These scores were the proportion of correct responses on the six social tasks, calculated by tallying the number of social tasks passed over the total number of social tasks (six). Similarly, the seven intrapersonal tasks were used to compute the composite “Intrapersonal ToM” score, as the reliability of these seven tasks was high (Cronbach's *α* = .70) and measured a variety of mental states (emotions, beliefs, and perception). These scores were the proportion of correct responses on the seven intrapersonal tasks, calculated by tallying the number of intrapersonal tasks passed over the total number of intrapersonal task taken (seven).

### 3.2. Correlations

See Table 3 for a zero-order correlation matrix for all measures. Social ToM was not significantly correlated to Intrapersonal ToM, *r* = .21, n.s.

### 3.3. Language and IQ Performance

To determine how children performed on the TOLD-I:4, the mean standard percentile score was computed for the full sample (*M* = 31.82, SD = 28.54), children with autism (*M* = 13.82, SD = 17.26), and children with Asperger syndrome (*M* = 53.38, SD = 23.18). An independent samples *t*-test showed that children with autism performed significantly lower than children with Asperger syndrome, *t*(71) = −8.50, *P* < .001. Due to this group difference, language was used as a covariate in many subsequent analyses.

To determine how children performed on the Raven's Progressive Matrices, the mean standard percentile score was computed for the full sample (*M* = 51.66, SD = 15.87), children with autism (*M* = 48.49, SD = 16.13), and children with Asperger syndrome (*M* = 55.29, SD = 14.97). An independent samples *t*-test showed no significant difference between children with autism and Asperger on this measure. 

To determine the relationship between language ability and IQ on ToM performance, Pearson's correlations were run between the Social and Intrapersonal ToM scores and the children's percentile scores on the TOLD-I:4 and Raven's Progressive Matrices. Performance on language measure was related to Social ToM (.62, *P* < .001) and Intrapersonal ToM (.55, *P* < .001). Similarly, performance on the IQ measure was related to Social ToM (.54, *P* < .001) and Intrapersonal ToM (.52, *P* < .001).

To investigate the contribution of language and IQ on ToM performance, a series of stepwise linear regressions was conducted. To determine if these regressions should be run on the full sample or separately for children with autism and Asperger syndrome, linear regressions were run with participant type, language percentile score (mean centered), and the interaction of participant type and language percentile score (mean centered) as predictors for Social and Intrapersonal ToM. Similarly, linear regressions were run with participant type, IQ percentile score (mean centered), and the interaction of participant type and IQ percentile score (mean centered) as predictors for Social ToM and Intrapersonal ToM. The interaction terms were not significant in any of these models, suggesting that the relationships between language and IQ on ToMs were not different for children with autism and Asperger syndrome. Therefore, the following stepwise linear regressions were conducted on the full sample. See Table 4.

To determine the contribution of language and IQ to Social ToM abilities, a stepwise linear regression was conducted for the full sample with Social ToM as the dependent variable. Language percentile score was entered in the first step. IQ percentile score was entered as a second step. Language accounted for 39% of the variance in Social ToM scores, *F*(1, 72) = 44.40, *P* < .001. IQ accounted for an additional 5% of the variance in Social ToM scores, *F*(1, 72) = 6.55, *P* < .05.

A stepwise linear regression was conducted for the full sample with Intrapersonal ToM as the dependent variable. Language percentile score was entered in the first step. IQ percentile score was entered as a second step. Language accounted for 33% of the variance in Intrapersonal ToM scores, *F*(1, 72) = 34.56, *P* < .001. IQ accounted for an additional 12% of the variance in Intrapersonal ToM scores, *F*(1, 72) = 15.52, *P* < .001.

### 3.4. Theory of Mind

**Full Sample**

**Figure 1:**
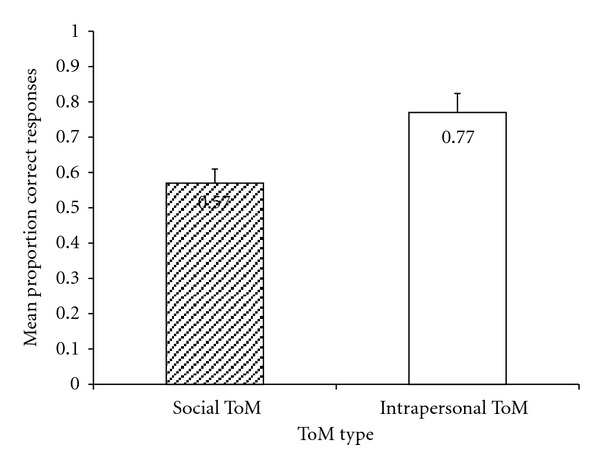
Mean Social and Intrapersonal ToM scores for full sample.

To determine if the composite Social ToM scores and Intrapersonal ToM scores were significantly different, a Repeated Measures ANCOVA was run with ToM Type (Social ToM, Intrapersonal ToM) as the within subjects factor and language as the covariate. A significant difference was found between the two composite scores, with children having a lower Social ToM score than Intrapersonal ToM score, *F*(1, 72) = 34.63, *P* < .01. See Figure 1.

**Group Differences**

To determine if Social ToM and Intrapersonal ToM scores varied as a function of participant group, a 2 × 2 Repeated Measures ANCOVA was run with ToM type (Social ToM, Intrapersonal ToM) as the within subjects factor, participant group (autism, Asperger) as the between subjects factor, and language as the covariate. Post hoc analyses were conducted to better understand the significant differences.

**Figure 2:**
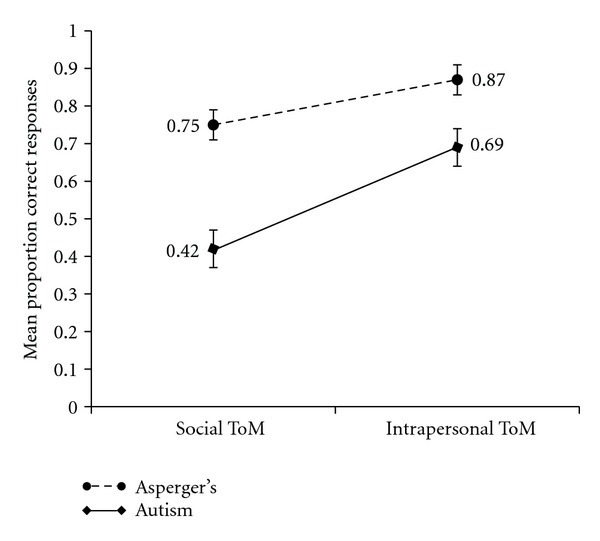
Mean proportion correct on Social ToM and Intrapersonal ToM for children with autism and Asperger syndrome.

Consistent with the Repeated Measures ANCOVA run on the full sample, the test revealed a significant main effect of ToM type, with children having higher Intrapersonal ToM scores, *F*(1,72) = 10.03, *P* < .05. See Figure 1. No significant main effect of participant type was found, *F*(1,72) = 1.49, *P* = .23. However, a significant interaction between participant type and ToM type was found, *F*(1,72) = 5.93, *P* < .05. The difference between Social ToM and Intrapersonal ToM scores was greater for children with autism than children with Asperger syndrome (*P* < .01). Post hoc analyses also revealed a significant difference between Social ToM scores for children with autism and Asperger syndrome, with children with Asperger syndrome having a higher Social ToM scores than children with autism (*P* < .05). No significant difference was found between the groups Intrapersonal ToM scores. See Figure 2.

## 4. Discussion

Three major issues related to ToM abilities in children with autism and Asperger syndrome were addressed. The first was whether ToM is a nonintegrated cognitive skill that differentiates into Social and Intrapersonal ToMs. The second was if Intrapersonal ToM was stronger than Social ToM in these populations. The third issue examined was whether Social and Intrapersonal ToM abilities were different for children with autism compared to children with Asperger syndrome.

### 4.1. ToM Differentiation

Findings showed that ToM differentiated. This result runs counter to the assumption that ToM is an integrated cognitive ability and, instead, lends support to differentiated accounts of ToM. Recent work has shown that other high-level cognitive abilities differentiate in children with Autism Spectrum Disorders, consonant with the current findings on ToM. For example, Williams and Happe [48] found that action monitoring differentiates and that children with autism are better able to recall their own actions than the actions of another.

For both groups of children in the current study, Intrapersonal ToM functioning was stronger than Social ToM performance. In fact, every child in the current study showed stronger Intrapersonal ToM than Social ToM. Social ToM performance was not strong, with the mean correct performance at 57%. These findings are concordant with both differentiated accounts of ToM. First, it makes sense when considering the functional uses of Social and Intrapersonal ToM as put forth by the FMS Model and the known impairments in autism and Asperger syndrome. The FMS Model proposes that Social ToM is used for social interaction [49] and children with autism and Asperger syndrome, by definition, have impaired social interactions [42]. On the other hand, Intrapersonal ToM is primarily used for learning [1, 49]. While it is true that many children with autism exhibit some learning difficulties, learning difficulties do not constitute a diagnostic criterion of autism. In fact, a recent study found that only thirty-five percent of children with autism have mental retardation [50]. Moreover, individuals with Asperger syndrome *cannot* possess a “clinically significant” cognitive delay by definition [42].

 The finding that Intrapersonal was stronger than Social ToM can also be used as support for the differentiated simulation account of ToM. Indeed, according to the simulation account intrapersonal reasoning is more advanced than social reasoning and is, in fact, necessary for the development of social reasoning skills. While the simulation account cannot explain previous findings that low-SES children exhibit stronger Social ToM than Intrapersonal ToM [2], the data from the current study fit the simulation account as well as the FMS Model.

### 4.2. ToM Differentiation by Group

While both groups exhibited weaker Social ToM than Intrapersonal ToM, this asymmetry was greater for children with autism, whose Social ToM function was especially weak (42% correct response rate). This finding makes sense when considering that Social ToM is proposed to play a role in social interactions and social interactions are more severely impaired in autism than Asperger syndrome. Hence, Social ToM development is vulnerable in children with autism because it is not heavily recruited or exercised.

 There was no difference in Intrapersonal ToM abilities across the two groups. This is not surprising when considering the function of Intrapersonal ToM as learning. The IQ scores of children with autism and Asperger syndrome were not statistically different, suggesting that learning potential was the same across groups.

The FMS Model proposes that reasoning about own and others' representations have distinct sociocultural bases. It may be that the nature of the disorders has lead to differential exposure to the relevant sociocultural experiences needed for Social and Intrapersonal ToM and, therefore, led to asymmetrical development across these forms of reasoning. The notion that different socialization experiences lead to different social and cognitive behaviors is not new (e.g., [51]). However, in this work it is being extended to ToM skills of children with autism and Asperger syndrome.

### 4.3. Language and Theory of Mind

It is important to note that language was highly correlated and contributed to ToM performance. This finding is consistent with the large body of literature documenting the relationship between ToM and language in typically developing children [52, 53], children with autism [54], and children with Asperger syndrome [32, 55, 56].

The current study contributes to the preexisting literature by suggesting that the relationship between language and ToM may be different based on function. Previous work investigating the relationship between language and ToM has been limited by relying nearly exclusively on performance on false belief tasks as a metarepresentational ToM measure (see [27]). By investigating ToM as a differentiated cognitive skill, the current study was able to show that language was slightly more strongly related to Social ToM than Intrapersonal ToM. Lucariello et al. [57] also investigated this possibility and found similar results; language accounted for more variance in ToM tasks that were social in nature than those that were object-oriented.

### 4.4. Limitations and Future Research

 A few limitations of the present research should be noted. First, no information was obtained regarding the amount and type of therapy or intervention participants received prior to participation. A variety of books and programs are available to help individuals with autism and Asperger syndrome develop (Social) ToM understanding [58–60]. It is possible that these resources were utilized more by one group than the other. If so, the group differences found in ToM skills may have been a reflection of intervention practices as opposed to the disorders themselves.

Moving forward, it will be important to explore if and how ToM differentiates among children of other populations. For example, by administering the measures used in the current study to typically developing children, one could determine the relative impairments seen in autism and Asperger syndrome. It would also be interesting to conduct comparative work with children who have language disorders but do *not* have autism or Asperger syndrome. Such work could clarify the impact language-versus-autism spectrum disorders have on Social and Intrapersonal ToM development. Further, it seems possible that asymmetrical ToM development may occur in a variety of disorders characterized by delays in social interaction and learning, as these skills are purported to use Social and Intrapersonal ToM, respectively. For example, it seems possible that those with disorders associated with social deficits such as schizophrenia, selective mutism, antisocial-, paranoid-, and narcissistic-personality disorders may exhibit unique Social ToM deficits. Similarly, it may be that those with learning related disorders, such as attention deficit hyperactivity, exhibit relatively weak Intrapersonal ToM. Findings from such studies would provide a deeper understanding of the disorders themselves, as well as help distinguish which of the two differentiated accounts best reflect the structure of ToM. 

 It is also possible that Social and Intrapersonal ToM tasks differ in ways in addition to the variation in target of the reasoning, such as memory demand. Every precaution was taken to eliminate this possibility. Standard, commonly-used ToM tasks were used and these were varied as little as possible to create Social and Intrapersonal versions. The only engineered difference between the Social and Intrapersonal versions of tasks was *the agent* specified in the questioning. For example, the unexpected contents task social condition question was “When Sally first saw the box, before *Sally* opened it, how did she feel about what was inside it?” The question form for the intrapersonal version was “When you first saw the box, before you opened it, how did you feel about what was inside it?” Hence, both questions entail memory—it is memory of *another's* past representation versus memory for *one's own *past representation that is compared. However, it may not be possible to rule out entirely that some unknown variable differed across tasks and influenced performance on the tasks.

## 5. Conclusions

The finding that ToM differentiates into Social and Intrapersonal ToM has important research and clinical implications. First, the definition of ToM may need to be reconsidered. This study contributes to a growing body of evidence showing that ToM is not a single integrated cognitive ability, but rather a differentiated cognitive construct based on target of the reasoning. The current study also provides a more thorough understanding of autism and Asperger syndrome. Both groups exhibited weaker Social ToM than Intrapersonal ToM. However, the children with autism exhibited a more severe Social ToM impairment than children with Asperger syndrome. This emerging understanding of autism and Asperger syndrome allows us to better understand these children. Moreover, this work can serve as a springboard for exploring novel treatment programs. Knowing that these children exhibit a differentiated ToM provides the opportunity to explore the effectiveness of interventions designed to capitalize on Intrapersonal ToM and perhaps utilize it as a pathway to develop Social ToM.

## Figures and Tables

**Table 1:** Predominant accounts of Theory of Mind.

ToM Account	Explanation of ToM development	References
*Integrated*		
Modularity	A module spontaneously processes attended actions, treats actions as intentional, and automatically computes mental states.	[22, 23]
Theory-theory	Children develop a theory of how the mind works and revise and replace that theory with successive theories.	[10, 24]
Sociocultural	Social contextual variables (e.g., interactions with friends and family) act as underlying sources of ToM development.	[5, 11, 25, 26]
Language	ToM develops from our ability to use language.	[27]

*Differentiated*		
Functional multilinear socialization	Reasoning about one's own and others' mental states are two distinct cognitive abilities: social reasoning and intrapersonal reasoning.	[1, 2]
Simulation	Children use self-reasoning skills to map and simulate other's reasoning.	[3, 4]

**Table 2:** Tasks and ToM behaviors by mental state assessed.

Task	Social	Intrapersonal
*Story Vignettes*. Story about a character (social) or participant (intrapersonal) that feels one emotion but depicts another	*Appearance-reality e* *mo* *ti* *on*.* “How does Diana/David really feel when ___? Does D/D feel happy or sad or okay?” “How does D/D try to look on her/his face? Does s/he look happy or sad or okay?”	*Appearance-reality e* *mo* *ti* *on*.* “How do you really feel when ___? Do you feel happy or sad or okay?” “How do you try to look on your face? Do you look happy or sad or okay?”

*Unexpected Contents*. SOCIAL: A band-aid box is opened to reveal crayons insideIntrapersonal: A closed toothpaste box is opened to reveal M&Ms inside	*Representational change emotion*.“When Sally/Sam first saw the box, before S/S opened it, how did s/he feel about what was inside it?” (sad)	*Representational change emotion*. “When you first saw the box, before you opened it, how did you feel about what was inside it?” (sad)
	*Representational change belief*. “When S/S first saw the box, before S/S opened it, what did s/he think was inside it?” (Band-Aids)	*Representational change belief*. “When you first saw the box, before you opened it, what did you think was inside it?” (toothpaste)
	*False belief*. “If another kid has not seen inside this box, when this kid first sees the box, before the kid opens it, what will the kid think is inside it?” (band-aids)	*Appearance-reality b* *el* *ie* *f*.* “What does it look like is in the box? (toothpaste) What is really in the box? (M & Ms)

*Unexpected Identity*. Deceptive object of a sponge looking like a rock is presented to view. Then, the child touches the object and its true identity is revealed.	*False belief*. “If another kid has not touched this and has not squeezed it, when this kid first sees it, before the kid touches it or squeezes it, what will the kid think it is?” (rock)	*Representational change belief*. “When you first saw this, before you touched it or squeezed it, what did you think it was?” (rock) *Appearance-reality belief*.* “What does this look like?” (rock) “What is this really?” (sponge)

*Color F* *il* *te* *rs*.* Filter placed over colored object such that only the child sees the color illusion.	*Appearance (for self)*. “You are looking at the cake with your eyes right now. Does it look green to you or does it look purple to you?” (green)	*Appearance (for self). * “You are looking at the butterfly with your eyes right now. Does it look blue to you or does it look pink to you?” (blue)
	*Perspective-taking perception * “I'm looking at the cake with my eyes right now. Does it look green to me or does it look purple to me?” (purple)	*Reality perception. * “What color is the butterfly really and truly? Is it really and truly blue or is it really and truly pink?” (pink)

*Both questions had to be answered correctly to receive a passing score.

**Table 3:** Zero-order correlations of all ToM measures.

			Theory of Mind item												
			Social						Intrapersonal						
			(1)	(2)	(3)	(4)	(5)	(6)	(7)	(8)	(9)	(10)	(11)	(12)	(13)
Theory of Mind item	Social	(1) SV App-real emotion	**1**	.350**	.256*	.497**	.516**	**.063**	.064	.054	.135	.215	.152	.145	.006
		(2) UC Rep-change emotion		**1**	.520**	.478**	.474**	.255*	.155	.315**	.260*	.304*	.176*	.206	.092
		(3) UC Rep-change belief			**1**	.834**	.514**	**.107**	.204	.200	.211	.212	.238*	.217	.015
		(4) UC false belief				**1**	.440**	.281*	.219	.298*	.297*	.184	.233*	.131	−.006
		(5) UI false belief					**1**	**.134**	.153	.178	.208	.132	.164	.195	.115
		(6) CF App-perception						**1**	.035	.181	.158	.273*	.204	.219	.370**
	Intrapersonal	(7) SV App-real emotion							** 1**	.278*	.219*	.346**	.371**	.319**	**.050**
		(8) UC Rep-change emotion								**1**	.524**	.344**	.465**	.264*	**.002**
		(9) UC Rep-change belief									**1**	.446**	.705**	.363**	.252*
		(10) UC App-real belief										**1**	.425**	.362**	.305**
		(11) UI Rep-Change Belief											**1**	.363**	.291*
		(12) UI App-real belief												**1**	.267*
		(13) CF App-reality													**1**

**Table 4:** Regressions of composite ToM scores on language and IQ.

Variable	Change in *R* ^2^	B (SE)	*β*	*R* ^2^
*Social ToM on language and IQ*				
Step 1				
Language^a^	.385***	.010 (.002)	.574***	.385
Step 2				
IQ^b^	.053*	.013 (.005)	.234*	.437
*Intrapersonal ToM on language and IQ*				
Step 1				
Language^a^	.327***	.008 (.001)	.502***	.327
Step 2				
IQ^b^	.122***	.017 (.004)	.356***	.449

Note. Betas are for the finals step in the model.

*P < .05.

***P < .001.

^a^TOLD:I-4 mean percentiles.

^b^Raven progressive matrices mean percentiles.
